# Structuring medication safety narratives: development and evaluation of the medication-related incident reports annotation scheme

**DOI:** 10.3389/fdgth.2026.1712526

**Published:** 2026-04-02

**Authors:** Huda Mohammad Alshammari, Denham L. Phipps, Elizabeth Le, Haifa Alrdahi, Penny J. Lewis, Riza Batista-Navarro

**Affiliations:** 1 Department of Clinical Pharmacy, College of Pharmacy, Northern Border University, Arar, Saudi Arabia; 2 School of Health Science, The University of Manchester, Manchester, United Kingdom; 3 Manchester University NHS Foundation Trust, Manchester, United Kingdom; 4 School of Computer Science, The University of Manchester, Manchester, United Kingdom; 5 Department of Computer Science, Faculty of Computers and Information Technology, University of Tabuk, Tabuk, Saudi Arabia

**Keywords:** medication safety, patient safety, incident reporting systems, natural language processing, annotation scheme, inter-annotator agreement, clinical text mining

## Abstract

**Introduction:**

Narrative reports of medication-related incidents contain valuable information about the causes and consequences of errors, but their unstructured format limits systematic analysis. Although natural language processing (NLP) can convert narrative reports into structured data, few annotation schemes have been developed specifically for medication safety and validated using real-world healthcare incident data. This study aimed to develop and evaluate the Medication-Related Incident Report Annotation (MRIRA) scheme, a multi-layer framework designed to structure narrative medication safety reports to support both qualitative analysis and automated text processing.

**Methods:**

Using narrative incident reports from the English National Health Service (NHS), a two-phase study design was implemented. In Phase 1, a purposive sample of 55 Controlled Drug incident reports was manually annotated to iteratively design the MRIRA scheme. The framework incorporated multiple annotation layers, including entities, events, attributes, and relations. The final scheme comprised 16 entity types, 11 event types, 5 attributes, 9 relation types, and 6 event argument roles. In Phase 2, two annotators independently applied the scheme to 30 incident reports, including 15 Controlled Drug reports and 15 reports from the National Reporting and Learning System/Learn from Patient Safety Events (NRLS/LFPSE). Inter-annotator agreement was evaluated using F1 scores under both strict and relaxed matching criteria.

**Results:**

Under strict evaluation, agreement was high for entity recognition (F1 = 0.85 and 0.91 across the two datasets) and entity–relation extraction (0.75 and 0.83). Agreement was moderate for event extraction (0.62 and 0.72) and acceptable for event attribute tagging (0.61 and 0.51). All metrics improved under relaxed matching criteria, indicating greater consistency when allowing minor boundary variation between annotations.

**Discussion:**

The MRIRA scheme provides a robust and reliable framework for structuring narrative medication safety reports. By enabling systematic extraction of entities, events, and contextual relationships from incident narratives, the scheme offers a high-quality annotated resource that can support the development of automated NLP tools and enhance organisational learning from medication-related incidents in healthcare systems

## 1 Introduction

Medication-related incidents remain a significant concern in healthcare systems worldwide. These incidents (which typically result from prescribing, dispensing or administration errors) not only pose risks to patient safety but also contribute to increased morbidity, prolonged hospitalisation, and healthcare costs (1, 2). In response to these challenges, incident reporting systems have been widely implemented across healthcare institutions as a mechanism for learning from incidents and preventing recurrence (3). These systems typically include predefined structured fields—for example, the type of medication error, stage of the medication-use process, severity of harm, and patient demographics—that enable standardised classification and basic trend analysis. Alongside these fields, they also capture free-text narratives, which are unstructured descriptions written by clinicians, pharmacists, or other staff at the point of care.

Narrative incident reports are often rich in contextual and causal information that is not captured in predefined categorical fields. They describe sequences of events, roles and responsibilities, system-level factors, and the reasoning behind actions taken (4–13). Despite their potential value, these narratives are notoriously difficult to analyse at scale due to their heterogeneity, variable length, diverse writing styles, and ambiguity in structure and terminology. Consequently, healthcare organisations often rely on manual review processes that are resource-intensive, inconsistently applied, and difficult to reproduce.

Recent advancements in Natural Language Processing (NLP) offer promising avenues for transforming unstructured clinical narratives into structured data that can support automated analysis, pattern recognition, and feedback into learning systems. NLP techniques such as Named-Entity Recognition (NER), relation extraction, and event detection have been applied to various clinical text domains, including electronic health records, pathology reports, and discharge summaries (14–18). However, incident report narratives differ in important ways from many other forms of clinical documentation. Unlike electronic health record entries, which are primarily designed to document clinical care processes, incident reports are retrospective accounts focused specifically on safety events. They are often written after the fact, may include subjective interpretation of causation, and frequently contain incomplete or conflicting information, reflecting their voluntary and explanatory nature (4, 9, 11, 12). Applying NLP to such narratives requires not only robust algorithms but also carefully constructed, domain-specific annotated corpora for training and evaluation.

In order to make more effective use of incident reports, there is a need to develop annotated corpora and annotation schemes tailored to incident reports. Zhang et al. (19) developed a corpus of Japanese medical incident reports annotated for intention (the intended action) and factuality (whether the action actually occurred), arguing that distinguishing between these is critical for understanding incident circumstances and supporting downstream NLP applications. Building on this work, Wong et al. validated their annotation scheme through a mixed-methods approach that included assessing inter-annotator agreement (IAA) (20). While these studies have advanced the field, limitations remain. Across the broader literature, many annotation schemes are restricted to narrow conceptual dimensions (e.g., focusing solely on error type), do not support multi-layered annotation such as relations between entities and events, fail to capture critical narrative details—particularly the connections between events and associated entities—or have not been systematically evaluated using real-world datasets (19, 21–25).

To be effective in such contexts, annotation schemes must also demonstrate reliability when applied by different annotators. IAA is a widely used measure of annotation reliability that quantifies the degree to which different annotators apply a scheme consistently to the same text, providing an important indicator of its clarity, usability, and reproducibility when applied to real-world datasets (20, 26–30). IAA is assessed using different metrics depending on the annotation task. For categorical labels, chance-corrected measures such as Cohen's kappa and Fleiss’ kappa are common (30, 31). For span-based tasks, such as identifying entities in free text, precision, recall, and F1 scores are preferred, as they capture both exact and partial matches (20, 32, 33). Using these metrics together provides a comprehensive assessment of annotation accuracy and consistency.

To address these gaps, the current study aimed to develop a multi-layered annotation framework for use with English language narrative reports of medication-related safety events.

## 2 Methods

### 2.1 Study design and overview

This study employed a two-phase design aimed at developing and evaluating a structured annotation scheme for medication-related incident reports. Phase 1 focused on the iterative development of the annotation scheme and the creation of a manually annotated corpus. Phase 2 evaluated the scheme's reliability through a formal IAA study using two independent annotators (H.M.A and E.L.).

The annotation scheme was designed to support structured representation of medication-related safety incidents, enabling both qualitative insight and downstream applications in NLP. All incident reports used in this study were drawn from two NHS England datasets: the Controlled Drug Reporting system and the Learning from Patient Safety Events (LFPSE) system (34–36). Reports from both systems were de-identified by the data holders before being shared for research use, and the research team conducted checks to confirm that no identifiable information remained. Ethical approval was obtained from the University of Manchester Research Ethics Committee [Reference ID 2023-15994], and data access permissions were granted under The University of Manchester's governance procedures.

### 2.2 Phase 1: Annotation scheme development

#### 2.2.1 Dataset selection and sampling

The datasets used in this study were obtained through formal data-sharing agreements with NHS England. Data were obtained from the NHS England Controlled Drug Reporting system, with access facilitated by the NHS England North West Controlled Drugs Team. This dataset comprised incidents relating to the prescribing, dispensing, administration, transcription, or monitoring of CDs. In the dataset, incident narratives were captured in free-text subsections designed to prompt reporters to provide contextual detail about the event. These subsections were labelled summary, underlying factors, actions taken, actions planned, and outcome (Table 1).

**Table 1:** Structure and definitions of narrative subsections in the CD incident reports dataset.

Data heading	Data type	Meaning
Summary	Free text	A brief overview of the incident.
Underlying	Free text	Factors contributing to the incident (e.g., short-staffed, busy environment).
Action Taken	Free text	Measures implemented to prevent recurrence.
Action Planned	Free text	Future plans to prevent similar incidents.
Outcome	Free text	Result of the incident (e.g., patient harm, professional retraining).

Phase 1. The CD dataset included 1,373 incidents reported in 2021–2022. From this extract, 400 reports (29%) were selected using a stratified systematic random sampling approach to create a screening pool. The dataset was first divided into four strata according to the reported level of harm (low, moderate, high, or unspecified). Within each stratum, one report was selected for every ten reports listed, ensuring representation across all categories while preserving randomness in selection. Of this screened sample, 98 reports (7%) were chosen for detailed annotation: 24 contained entries across all narrative subsections, while 74 were included on the basis of extended narratives (>100 words). This ensured both representation across harm levels and narrative richness.

The proportion of reports selected for annotation (98/1,373; 7%) was determined based on the methodological requirements of annotation scheme development rather than to estimate the prevalence of medication error types across the NHS. The objective at this stage was to ensure sufficient variation in harm level, medication-use stage, and narrative structure to support iterative refinement of labels and definitions. Reports were reviewed in batches, and modifications to the scheme became progressively minor and clarification-focused over time, indicating convergence of the scheme structure. The selected sample therefore provided sufficient conceptual and narrative diversity to establish coherent entity, event, attribute, and relation categories before proceeding to formal reliability evaluation. While larger datasets may reveal additional rare patterns, the development sample was appropriate for the intended methodological purpose.

#### 2.2.2 Annotation approach and iterative development

Annotation followed a bottom-up inductive approach, grounded in close reading and empirical observation of the narrative content. The first author (H.M.A.), a clinical pharmacist with expertise in medication safety, conducted all initial annotations using a draft scheme informed by a literature review of previous work on medication error classification, patient safety taxonomies, insights from stakeholders, and narrative analysis.

Reports were annotated in batches of 5–10. After each batch, annotation-related issues—such as ambiguities in label definitions, overlapping constructs, or difficulties with rare and nested events—were logged, and the scheme was revised to improve conceptual clarity, granularity, and applicability. These issues were recorded and discussed with the research team, and the reports were then reannotated to ensure consistency with the updated scheme. This process continued until conceptual saturation was reached (55 CD reports) and no further labels or revisions were required.

### 2.3 Annotation tool

Annotation was performed using the BRAT Rapid Annotation Tool (BRAT), an open-source, web-based platform commonly used in clinical and biomedical NLP tasks (37, 38). BRAT was selected for its ability to support multi-layer annotation, including entities, events, attributes, and relations.

**Figure 1:**
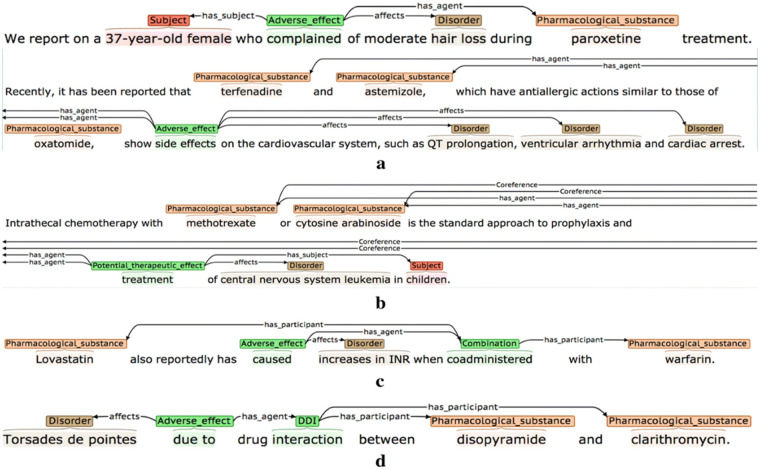
Examples of event annotations for different event types (the semantic types of the events are **(a)** Adverse effect, **(b)** Adverse effect, **(c)** Combination, and **(d)** Drug-drug interaction), reproduced from Thompson et al. (18), licensed under CC BY 4.

Incident reports were converted into plain-text format and uploaded into BRAT's environment. The tool allows an annotator to highlight spans of interest, assign type-specific labels, define directional relations between spans, and tag additional semantic attributes (e.g., negation). Annotated files were saved in BRAT's standoff format (.ann and.txt), enabling conversion into structured formats compatible with machine learning (ML) pipelines. To illustrate the annotation environment, Figure 1 presents a screenshot of BRAT showing an example sentence with highlighted entities, linked relations, and event labels.

The tool's visual interface provided real-time feedback on label consistency and allowed for quick navigation across long narratives. It also enabled reannotation of reports following guideline updates, ensuring consistency in final annotations.

### 2.4 Annotation procedure

No pre-processing, tokenisation, or automated text segmentation was performed before annotation; all reports were annotated in their original narrative form. Each report underwent detailed manual annotation by the first author. The annotation process involved the following steps:

- Text Preparation: Reports were converted to.txt format and uploaded into BRAT (38).
- Initial Review: The annotator read the narrative line by line to familiarise themselves with the content.
- Entity and Event Annotation: Relevant spans were marked with appropriate labels from the entity and event sets (see Table 2 for an overview of the scheme's entity and event types) (18, 19, 39).
- Relation Annotation: Links between entities and between events and entities (e.g., drug–dose, event–drug) were annotated using pre-identified directional relation types and argument roles (examples shown in Figure 1 and see Table 2 for relation types) (18, 39).
- Attribute Tagging: Events were examined for modifying features such as negation, intent and actuality. These were tagged using attribute labels (Table 2).
- Ambiguity Logging: Difficult or unclear cases were recorded in an issue log, with rationale and alternative interpretations.
- Revision and Reannotation: Upon updates to the annotation scheme, previously annotated reports were revisited to ensure compliance with current definitions.

**Table 2:** Overview of the MRIRA scheme's entity and event types and other categories.

Category		Annotation labels
Entity types	General	‘People’, ‘Location’, ‘Date/Time’, ‘Artefact’, ‘Knowledge’, ‘Function’
	Patient-specific	‘Age group’, ‘Gender’
	Domain-specific	‘Drug name’, ‘Dosage form’, ’Strength or amount’, ‘Dose’, ‘Frequency’, ‘Route of administration’, ‘Duration’, ‘Medical condition’
Entity relations		‘Has dose’, ‘Has frequency’, ‘Has form’, ‘Has route’, ‘Has duration’, ‘Has strength or amount’, ‘Has time’, ‘Has', ‘at’
Main event types		‘Prescribing’, ‘Transcription’, ‘Dispensing’, ‘Administration’, ‘Monitoring’
Supplementary event types		‘Corrective action’, ‘Preventive action’, ‘Underlying and contributing factors’, ‘Error outcome’, ‘Action taken’, ‘Other action’
Event argument roles		‘Agent’, ’Subject’, ‘Receiver’, ‘When’, ‘Where’, ‘For’
Event attributes		‘Action intended and actual’, ‘Action not intended but actual’, ‘Action intended but not actual’, ‘Action intent not clear’, ‘Negated’

### 2.5 Phase 2: Inter-annotator agreement study

#### 2.5.1 Dataset and annotators

To evaluate the consistency of the annotation scheme across users, a subset of 30 incident reports was selected for independent annotation by two annotators. From the 55 CD reports annotated in Phase 1, a subset of 15 (approximately one-quarter) was selected to ensure a manageable workload while still representing the diversity of cases. To enable cross-dataset comparison, an equal number of NRLS/LFPSE reports (*n* = 15) was added, giving a total of 30. This subset included:

- 15 reports from the CD dataset
- 15 reports from the NRLS/LFPSE database

Reports were chosen on the basis of narrative richness, enabling assessment of the annotation scheme's generalisability beyond its original CD development context and across different reporting systems within the NHS. This design allowed assessment of the scheme's generalisability across two structurally different datasets.

The datasets used in this study were obtained through formal data-sharing agreements with NHS England. While the CD dataset comprised incidents specifically relating to CDs, the NRLS—now superseded by the LFPSE service—contained a wider range of medication-related incidents. Similar to the CD dataset, NRLS/LFPSE narratives were captured in free-text subsections designed to prompt reporters to provide contextual detail about the event. The NRLS/LFPSE dataset used different headings but collected equivalent information to the CD dataset, for example: description of what happened (similar to summary), apparent causes (similar to underlying factors), and actions to prevent recurrence (covering elements of actions taken and actions planned). These parallels supported the consistent application of the annotation framework across both datasets.

The two annotators were the doctoral researcher (H.M.A.), who developed the MRIRA scheme and conducted the initial Phase 1 annotations, and a foundation doctor (E.L.) with PhD-level training in ML and prior research experience in NLP. Before commencing independent annotation, the second annotator underwent structured training that included one-on-one briefing sessions, a detailed review of the annotation guidelines, practice annotations on sample reports, and feedback discussions to resolve inconsistencies. Both annotators then worked independently and were blinded to each other's annotations. After completing the annotation of the study subset, they met to review and discuss areas of disagreement, clarify guideline ambiguities, and update their annotations independently where required (26).

#### 2.5.2 Inter-annotator agreement metrics

Agreement was assessed across four annotation layers: entity annotation, relation extraction, event annotation, and attribute tagging. IAA was measured using precision, recall, and F1 scores, with one annotator (H.M.A.) designated as the reference (i.e., ground truth) and the other (E.L.) as the response. Precision represented the proportion of the response annotator's labels matching the reference, while recall represented the proportion of relevant labels in the reference identified by the response. The F1 score, calculated as the harmonic mean of precision and recall, was chosen for its balanced evaluation of annotation quality, particularly in complex entity and event classification tasks (26, 39).

For each layer, scores were computed under two matching conditions:

- Strict matching – both span boundaries and the assigned label had to match exactly (40).
- Relaxed matching – partial overlap of spans was permitted, provided the label matched (40).

F1 score interpretation varies by task complexity and domain; in NER, scores above 0.80 are considered high, 0.60–0.79 moderate, and below 0.60 low, while event annotation often yields lower scores, with 0.50–0.60 potentially acceptable (22, 25, 26, 32, 40). All metrics were calculated automatically using custom Python scripts (Available upon request) to ensure consistency and transparency. Scores were reported separately for each annotation layer and each dataset (CD and NRLS/LFPSE), enabling cross-layer and cross-dataset comparison.

## 3 Results

### 3.1 Phase 1: Development of the MRIRA scheme and annotated corpus

#### 3.1.1 MRIRA scheme

The outcome of Phase 1 was the creation of a conceptually grounded, multi-layer annotation scheme for structuring medication-related incident narratives, termed the Medication-Related Incident Report Annotation (MRIRA) scheme. The final MRIRA scheme comprised 16 entity types, 11 event types, 5 attribute types, 9 relation types, and 6 event argument roles (see Table 2 for an overview and Table 3 for relation types). Full descriptions of scheme types with illustrative examples are provided in the supplementary tables (Supplementary Tables S1–S3).

**Table 3:** Entity relation types of the MRIRA scheme.

Relation type	Source entity type	Target entity type
has dose	‘Drug name’	‘Drug dose’
has frequency	‘Drug dose’	‘Drug frequency’
has frequency	‘Drug name’	‘Drug frequency’
has form	‘Drug name’	‘Drug form’
has route	‘Drug name’	‘Dose route’
has duration	‘Drug name’	‘Dose duration’
has strength or amount	‘Drug name’	‘Drug strength or amount’
has time	‘Drug dose’	‘Date/Time’
has	‘People’	‘Medical condition’
at	‘People’	‘Location’

Each type was defined with a precise label definition, annotation rules specifying when to apply the label, clear inclusion/exclusion criteria, and examples drawn from the training corpus. The guidelines explicitly described span boundaries and differentiation rules for overlapping types. The complete MRIRA guidelines are available from the corresponding author upon reasonable request.

For example, the ‘Prescribing’ event was defined as any documented or verbally given decision to initiate, discontinue, or adjust a medication order. The ‘Negated’ attribute was applied to modify an event label when the narrative indicated that the event did not occur. For example, in the sentence “The dose was not given”, the action would first be annotated as an ‘Administration’ event to reflect the type of medication-related action involved. The ‘Negated’ attribute would then be attached to this event to specify that the administration did not take place. These dual labelling captures both the semantic type of action (administration) and the fact that it was explicitly negated in the report.

The scheme was then applied to a subset of incident reports to produce a fully annotated reference corpus, described in Section 3.1.2.

#### 3.1.2 Final annotated corpus

**Figure 2:**
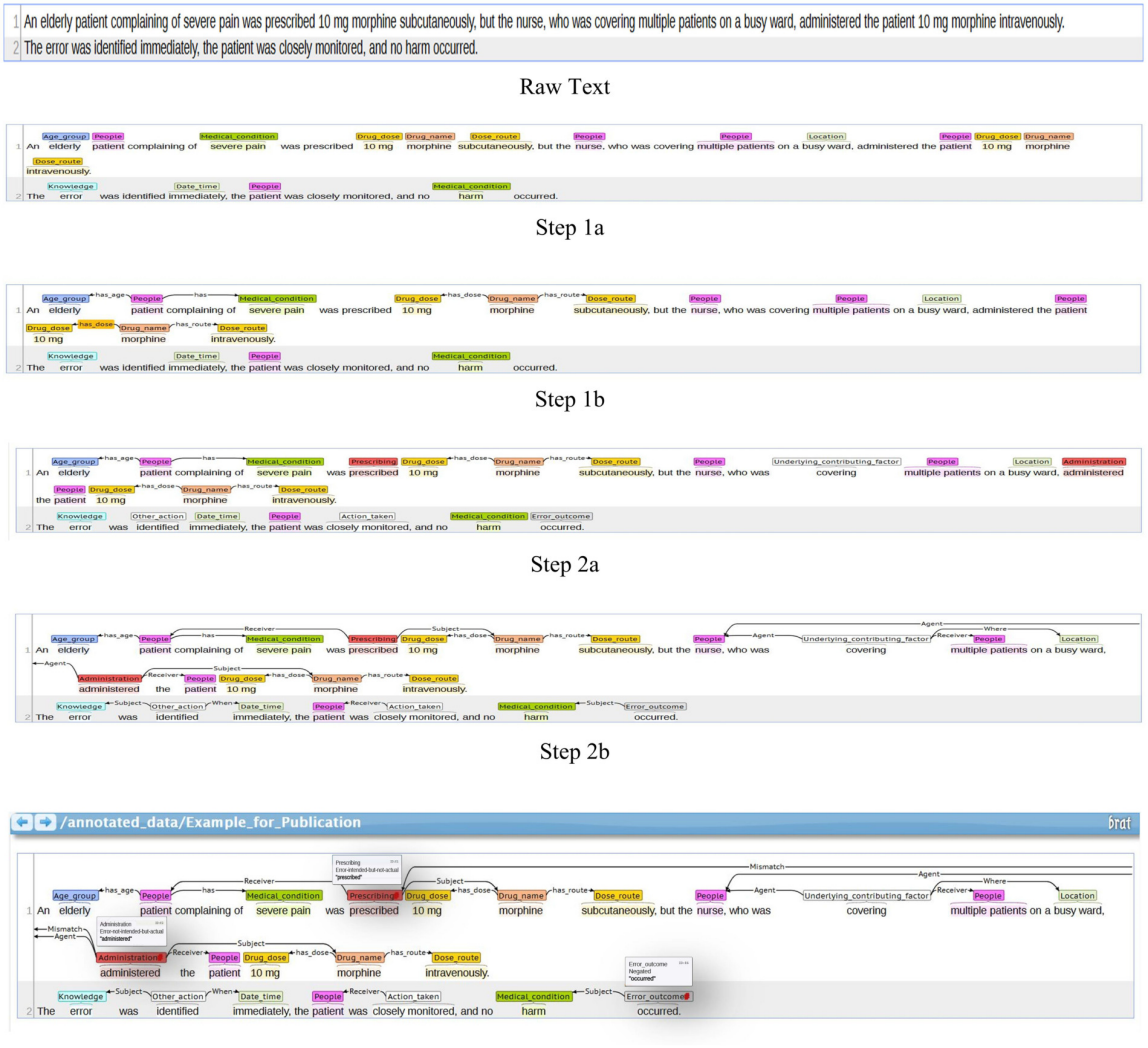
Step-by-step annotation of an example incident narrative using the MRIRA scheme.

The final annotated corpus from Phase 1 consisted of 55 fully annotated CD reports. Each report was annotated with one or more entities, events, attributes, and relations, covering different incident types. In total, the corpus contained over 700 annotated events, approximately 1,700 entity mentions, more than 50 attribute annotations, and several hundred relations linking clinical and general concepts (e.g., ‘Medical condition’ linked to ‘People’) and general concepts (e.g., ‘Age group’ linked to ‘People’). To aid reader understanding, we provide a running example in Figure 2, which shows how a raw sentence is incrementally annotated across layers: entities, events, relations, and attributes. The annotation process is illustrated below, beginning with the raw sentence and progressing through each annotation layer.

To illustrate this stepwise process in practice, Figure 2 presents a worked example showing how a single incident narrative is progressively annotated across the MRIRA layers, with the detailed explanation provided in the following text.

Step 1a – Entities identification

- Age group → *elderly*
- People → *patient*, *nurse*, *multiple patients*
- Medical condition → *severe pain*, *harm*
- Drug dose → *10 mg*
- Drug name → *morphine*
- Dose route → *subcutaneously*, *intravenously*
- Location → *busy ward*
- Date/Time → *immediately*
- Artefact (Knowledge) → *error*

Step 1b – Relations

- Patient (People) → has_age → Age group (elderly)
- Patient (People) → has medical condition → severe pain
- Morphine (Drug name) → has_dose → 10 mg
- Morphine (Drug name) → has_route → subcutaneously, intravenously

Step 2a – Event triggers

- Main event → Prescribing (*prescribed)*, Administration (*administered)*
- Supplementary action → Underlying contributing factor (*covering),* Other action (*identified*), Action taken (*monitored)*, Error outcome (*occurred)*

Step 2b – Event arguments

- Prescribing → Subject = morphine; Receiver = patient
- Administration → Subject = morphine; Agent=nurse; Receiver = patient
- Underlying factor (covering) → Agent = nurse; Receiver = multiple patients; Where = busy ward
- Other action (*identified*) → Subject = error; When = immediately
- Action taken (*monitored*) → Receiver = patient
- Error outcome (*occurred*) → Subject = harm

Step 2c – Attributes

- Prescribing: Error-intended-but-not-actual
- Administration: Error-not-intended-but-actual
- Error outcome: Negated

This corpus provided the empirical basis for scheme refinement and served as a reference dataset for subsequent evaluation. It captured a wide range of entities, events, and relations, offering a structured representation of medication safety narratives. Frequently annotated entities included ‘People’ (335 instances), ‘Drug name’ (194), and temporal references (‘Date or time’, 129), while less frequent categories such as ‘Medical condition’ (26) and patient demographics appeared only occasionally.

Among the main events, ‘Administration’ (50) and ‘Prescribing’ (42) were the most frequent, reflecting the prominence of these stages in reported incidents, whereas ‘Transcription’ (2) and ‘Monitoring’ (0) were rare or absent. Supplementary events such as ‘Corrective action’ (144) and ‘Preventive action’ (67) were highly frequent, underscoring the strong emphasis on documenting follow-up measures and prevention efforts in incident reporting.

Event arguments such as ’Subject’ (247), ‘Receiver’ (139), and ‘Agent’ (73) were also widely represented, highlighting the scheme's ability to capture who was involved, what was affected, and when and where incidents occurred. Attributes such as intention/actuality, mismatch, and negation were less frequent but provided valuable detail for distinguishing subtle narrative features.

Together, these corpus characteristics demonstrate both the narrative diversity of CD incident reports and the capacity of the MRIRA scheme to capture common and rare aspects of medication safety data. Some annotation categories demonstrated low or zero frequency within the development corpus (e.g., ‘Monitoring’, ‘Dose duration’). This reflects the bounded size and contextual focus of the sampled dataset rather than structural redundancy within the MRIRA framework. Rare but safety-relevant types were intentionally retained to preserve conceptual completeness and future extensibility. Application of MRIRA to larger and more heterogeneous datasets will allow further empirical evaluation of the distribution and frequency of these less common categories. A full statistical overview of the MRIRA corpus is presented in Table 4.

**Table 4:** Statistics of the MRIRA corpus for 55 CD incident reports.

Annotation level	Annotation type	Number of annotations	Average words per annotation	Total annotation
Entities	‘Drug name’	194	1	247
	‘Drug strength or amount’	52	2	84
	‘Drug form’	55	1	62
	‘Drug dose’	66	1	74
	‘Dose frequency’	26	2	47
	‘Dose route’	5	1	5
	‘Dose duration’	0	0	0
	‘People’	335	1	485
	‘Location’	58	2	98
	‘Artefact’	110	2	178
	‘Knowledge’	84	2	194
	‘Function’	50	2	118
	‘Date/Time’	129	2	275
	‘Medical condition’	26	2	53
	‘Patient age’	1	3	3
	‘Patient gender: female’	4	1	4
	‘Patient gender: male’	5	1	5
	‘Patient gender: other’	0	0	0
Event	‘Prescribing’	42	1	62
	‘Transcription’	2	2	5
	‘Dispensing’	10	1	12
	‘Administration’	50	1	69
	‘Monitoring’	0	0	0
	‘Corrective action’	144	1	161
	‘Preventive action’	67	2	141
	‘Underlying or contributing factor’	16	2	37
	‘Error outcome’	11	3	32
	‘Other actions’	8	1	8
Event arguments	‘Agent’	73		
	‘Subject’	247		
	‘Receiver’	139		
	‘Where’	15		
	‘When’	58		
Events attribute	Intent and actuality annotation	20		
	Mismatched events	13		
	‘Negated’	5		

#### 3.1.3 Annotation challenges and iterative refinement

The annotation process during Phase 1 revealed several domain-specific challenges that informed the evolution of the scheme. One challenge was ambiguity in event boundaries, as some reports contained long or nested events—such as multiple errors described within the same sentence—requiring precise guidance on span selection.

Another challenge was the presence of implicit content, where the type of error was not explicitly stated. For example, in “*The nurse noticed the wrong dose before administration*”, it is unclear whether the wrong dose arose from a prescribing error, a preparation/dispensing error, or an imminent administration error. Such cases required explicit guidance on how to annotate when the underlying error type could only be inferred.

Fragmented or incomplete sentence structures were another challenge. Many incident descriptions did not follow complete sentence syntax, which made annotation—particularly event extraction—more difficult. In such cases, it was often challenging to identify a clear event trigger word and accurately link it to its arguments (e.g., the agent, subject, and receiver). For instance, “*Incorrect dose – patient not harmed*” contains two events: the main event (“*Incorrect dose*”), where the type of error and trigger word are absent, and the supplementary event (“*patient not harmed*”), which represents the outcome but lacks a clear action verb. Both require interpretation before event and argument annotation can be applied consistently. To address these issues, the guidelines were updated iteratively, with examples added to clarify span boundaries, attribute application, and relation construction.

### 3.2 Phase 2: Inter-annotator agreement study

#### 3.2.1 Dataset characteristics

The IAA study involved 30 reports, split evenly across the two datasets: 15 CD reports and 15 NRLS/LFPSE reports.

The CD reports were generally shorter, with concise narrative structure, while NRLS/LFPSE reports varied more in length and linguistic complexity. This dual-sourcing allowed the scheme's reliability to be tested across different incident reporting formats and scope within the NHS.

#### 3.2.2 Overall inter-annotator agreement results

The results of the IAA evaluation are summarised in Tables 5, 6 below.

**Table 5:** Averaged F1 scores by annotation layer for CD incident reports.

Annotation Layer	Strict F1 Score	Relaxed F1 Score
Entities	0.85	0.92
Relations	0.75	0.85
Events	0.62	0.75
Attributes	0.61	0.65

**Table 6:** Averaged F1 scores by annotation layer for NRLS/LFPSE reports.

Annotation Layer	Strict F1 Score	Relaxed F1 Score
Entities	0.91	0.94
Relations	0.83	0.87
Events	0.72	0.8
Attributes	0.51	0.53

In addition to F1 scores, precision and recall were examined to further characterise disagreement patterns. Across most annotation layers, precision and recall were broadly comparable, indicating no systematic tendency toward over-annotation or under-annotation by either annotator. Where differences were observed, particularly in attribute tagging, variability was more often associated with recall (i.e., missed labels) than with reduced precision. Detailed precision and recall values are available from the corresponding author upon reasonable request.

Across all layers and datasets, relaxed evaluation produced higher scores than strict evaluation. Entity and event layers showed the highest agreement, particularly under relaxed evaluation, suggesting a shared understanding of core concepts such as ‘Drug name’, ‘Prescribing’, and ‘Date or time’. Attributes displayed greater variability across datasets, reflecting their interpretive complexity and indicating that the MRIRA scheme may benefit from enhanced guidance or decision rules for tasks such as event attribute tagging. CD reports were generally shorter and followed a more standardised regulatory structure, reflecting the formal governance framework surrounding CD reporting in England. However, for all annotation layers except event attribute annotation, agreement scores were higher for NRLS/LFPSE reports than for CD reports.

#### 3.2.3 Common disagreements and qualitative insights

Review of disagreement cases revealed several recurring themes. One is *span granularity* — annotators differed in how much text to include within a span; for example, one selected “morphine sulfate” as ‘Drug name’ while the other selected only “morphine”. Relaxed evaluation helps to overcome this disagreement, which contributes to a less strict evaluation result. Another is *attribute assignment* — disagreements often arose from a missing attribute on one side or from applying the attribute to different events; for example, one annotator marked the ‘Administration’ event as ‘Intended–not actual’ for “the evening dose was intended but not given”, while the other omitted the attribute. Similarly, one annotator applied ‘Negated’ to ‘Outcome’ (“harm”) in “no patient harm reported” while the other left the attribute unset. Third is *relation scope* — annotators sometimes linked events to different entities (e.g., “Wrong dose administered” connect ‘Administration’ to the ‘Drug name’ not to ‘Drug dose’ as ‘object’ of the event, chose different ‘has-dose’/‘has-route’ links, or omitted a ‘Mismatch’ relation where text implied a discrepancy (e.g., “prescribed oral, administered IV”). It is also possible that prior exposure to the scheme and its application to CD reports contributed to higher scores when annotating NRLS/LFPSE reports, as these were completed later in the evaluation process.

These observations highlight the need for guideline refinements around span boundary definition, consistent application of action intent and ‘Negated’, and cross-sentence linking (including ‘Mismatch’ and has-relations). Sufficient training and pilot annotation are also important to ensure annotators have adequate exposure to the tasks before formal evaluation.

## 4 Discussion

This study presents the development and evaluation of the MRIRA scheme, a multi-layered framework designed to structure narrative data from medication-related incident reports. Developed iteratively using real-world data from the NHS CD reporting system and evaluated for reliability through a formal IAA study on both CD and NRLS/LFPSE datasets, the MRIRA scheme demonstrated both conceptual robustness and practical applicability across distinct reporting scopes and formats.

The resulting annotated corpus not only supported iterative refinement of the scheme but also provides a structured benchmark resource for future NLP development and comparative studies in medication safety text analytics.

A key strength of this study is its bottom-up, iterative development approach, grounded in detailed manual annotation of real-world reports. This method allowed for the progressive refinement of labels based on actual narrative patterns rather than theoretical assumptions alone. By selecting reports that reflected a wide range of medication-related incidents, the scheme was able to capture both common and rare features of safety narratives—ensuring broad conceptual coverage.

Another strength was the scheme's multi-layered structure, which captures not only entities and the main event but also supplementary details such as contributory actions, corrective actions, and preventive actions, along with relevant attributes (e.g., negation, intended-not-actual) and relations (e.g., temporal or contextual links, mismatches). This layered design enhances the scheme's expressive power and allows for more nuanced modelling of safety incidents. While this complexity introduced some annotation challenges, as shown by lower relation agreement, it was necessary for capturing the full richness of incident narratives. In contrast to earlier single-label schemes that oversimplify narratives into static categories, MRIRA supports dynamic analysis of sequences, causes, and consequences within incidents. Its emphasis on relation extraction enables reconstruction of cause–effect chains, linking drugs to doses or routes, and identifying gaps or mismatches in action sequences. Few other published schemes in this domain attempt this level of structural modelling, positioning MRIRA as a novel contribution to the field.

MRIRA build upon previous studies, such as Zhang et al. (19) and Wong et al. (20), by adopting a more modular, multi-layered structure encompassing entities, events, attributes, arguments, and relations. It supports the reconstruction of event sequences and connections, providing insight into how incidents evolve across the medication-use process. Developed from English-language NHS CD reports and evaluated on both CD and NRLS/LFPSE reports, MRIRA is aligned with the terminology, style, and structure of UK safety narratives. These design features make it a more modular and extensible framework for both clinical and general concepts mentioned in the report text, and therefore better suited for large-scale narrative analysis and NLP-driven safety improvement.

The MRIRA scheme demonstrated strong IAA for entities and their relation annotations, moderate agreement for events, and lower agreement for attributes. This profile is consistent with prior annotation research, where entity-level tasks are generally more reliable than interpretative layers. Pustejovsky & Stubbs (22) note that entities are relatively straightforward to annotate, while events and attributes require greater interpretative judgement. To reinforce this point, Roberts et al. (23) likewise reported higher reliability for entities compared with events and relations, and similar patterns have been observed in more recent studies. For instance, Salek Faramarzi et al. (41), Schäfer et al. (42), Raza & Schwartz (43), and Wong et al. (21) all found that entities achieve stronger agreement than events, relations, or attributes. Collectively, these findings confirm that MRIRA follows a well-established pattern in patient safety and clinical NLP domains, supporting its reliability and reproducibility.

However, not all studies report the same distribution of reliability across annotation layers. Contrasting evidence shows that interpretative dimensions can achieve reliability equal to or even higher than entities under certain conditions. Zhang et al. (19) and Wong et al. (20) both reported strong agreement for entities and attributes, while Zhu et al. (44) likewise found entities and attributes to be reliably annotated but relations less so. Uzuner et al. (24) also reported assertion classification (attributes) as the easiest task, concept extraction (entities) as more complex, and relation classification as the most challenging overall. This contrasts with MRIRA, where attributes were the least reliable, though all studies converge in showing that relations are consistently more difficult than entities. These divergences highlight the influence of dataset scale, scope, and complexity in shaping annotation reliability.

The variation between Zhang, Wong, and Wong is particularly instructive, as all three applied the same intention/factuality schema yet produced different results (19–21). Zhang (19) and Wong (20) reported high attribute reliability, whereas Wong (21), applying the schema to over 58,000 heterogeneous reports, observed substantially lower attribute performance, closely aligned with MRIRA. This contrast suggests that MRIRA's attribute reliability reflects the inherent complexity of NHS narratives rather than a weakness of the scheme itself.

Importantly, the high relaxed F1 scores across all MRIRA annotation layers support the interpretation that many disagreements were minor, such as differences in span boundaries—rather than major conceptual divergence. This finding reinforces that the scheme can be applied consistently with appropriate training, and that inconsistencies can be mitigated through guideline refinements and structured feedback during annotator calibration. As in other multi-axis frameworks [e.g. (45, 46)], attributes and relations remain the most demanding layers. Lower agreement in these areas reflects the inherent complexity of the task, underscoring the importance of clear guidelines, decision rules, and targeted training in sustaining annotation reliability.

The use of two datasets (CD and NRLS/LFPSE) added further rigour by testing the scheme's applicability across different narrative formats and reporting styles. The consistency in agreement across datasets therefore provides early evidence of the scheme's generalisability to other NHS safety reporting systems.

The development of MRIRA and its validated application to real-world incident reports provides a foundational resource for both research and practice. The annotated corpus offers high-quality training and evaluation data for supervised NLP models aimed at detecting medication safety concepts, while the scheme itself can be adapted into annotation templates or prompts for semi-automated systems, including AI-assisted incident reporting and clinical decision support tools. By converting unstructured narratives into structured, analysable data, MRIRA supports both qualitative insight and quantitative scalability, enabling the identification of patterns—such as recurring contributory actions, staff roles, or outcome types—that can be used to triage and prioritise incidents, allowing safety teams to focus on high-risk cases. Applied at scale, the scheme could underpin dashboards of annotated insights, strengthening feedback loops often missing from traditional incident learning systems. Beyond retrospective analysis, MRIRA's design also lays the groundwork for future applications, including automated error detection, targeted feedback generation, and AI-assisted safety surveillance.

Although the primary aim of MRIRA was to structure narrative data for semantic modelling, the annotated outputs inherently support quantitative analysis. Each annotated entity, event, attribute, and relation constitutes a discrete structured unit that can be computationally aggregated. When applied at scale, the framework enables frequency analysis of medication error stages (e.g., ‘Prescribing’, ‘Administration’), contributory factors, corrective actions, and outcomes, thereby supporting distributional modelling alongside qualitative interpretation. While the present study did not conduct epidemiological quantification across the full dataset, the architecture of MRIRA enables systematic estimation of event frequency and pattern distribution in future large-scale implementations.

While these applications highlight the scheme's potential, several limitations must be acknowledged.

First, the development corpus was limited to a subset of 98 reports, with 55 fully annotated during iterative refinement. Although sufficient to achieve conceptual saturation for scheme development, this sample size may not fully represent the variability of medication-related incidents across the NHS or other healthcare systems. Rare annotation types may therefore be underrepresented, and larger-scale application may reveal additional distributional patterns. In addition, the development phase relied exclusively on CD reports. As CDs are subject to stricter regulatory oversight and governance requirements within the UK, the type, structure, and level of detail in these reports may differ from those involving non-controlled medications. This may have influenced the prominence of certain event types, corrective actions, or contributory factors within the development corpus. Although cross-dataset evaluation partially mitigates this concern, future development using broader medication categories would further strengthen representativeness.

Second, the inter-annotator agreement evaluation involved a single second annotator. While this approach is consistent with many annotation scheme development studies and yielded robust F1 scores, inclusion of multiple independent annotators would allow more comprehensive reliability estimation and formal statistical comparison across annotator pairs.

Third, the datasets were derived exclusively from NHS England reporting systems. Reporting cultures, terminology usage, and system structures may differ internationally, which may limit generalisability beyond the UK context. Validation of MRIRA in other healthcare systems will therefore be important to assess transferability.

Fourth, although MRIRA was designed to support downstream NLP development, this study did not implement or benchmark a machine learning model trained on the corpus. Automated extraction performance and scalability remain to be empirically evaluated in future work.

Finally, the iterative development process relied primarily on the expertise of a single primary annotator during Phase 1. While this facilitated conceptual coherence, broader collaborative guideline refinement may further strengthen robustness and external validity.

## 5 Conclusion

This study presents the development and evaluation of the Medication-Related Incident Report Annotation (MRIRA) scheme, a structured, multi-layer framework for analysing free-text narratives in medication-related incident reports. Iteratively designed using English NHS Controlled Drug reports and validated across two reporting systems, MRIRA captures both high-frequency and safety-critical low-frequency features with strong inter-annotator agreement for entities and events. It provides a comprehensive annotation structure, an annotated NHS corpus for NLP model development, and empirical reliability evidence to support large-scale narrative analysis and automation. By enabling fine-grained representation of events, entities, attributes, and their interrelationships, MRIRA bridges the gap between qualitative insight and computational scalability in medication safety. Future work will expand the annotated corpus across multiple NHS trusts and additional medication domains to enhance representativeness and statistical robustness. Integration with supervised and transformer-based NLP models will allow benchmarking of automated extraction performance against validated manual annotations. Multi-site validation studies are also needed to examine cross-context generalisability and to evaluate whether structured narrative extraction improves organisational learning outcomes, feedback timeliness, and safety intervention design within routine patient safety reporting systems.

## Data Availability

The data analyzed in this study is subject to the following licenses/restrictions: due to NHS confidentiality and patient safety restrictions, the annotated datasets used in this study cannot be shared. However, the MRIRA scheme and the Python codes developed for calculating IAA are available from the corresponding author upon reasonable request. Requests to access these datasets should be directed to dr.huda.m@outlook.sa.
