# Supplementary material for: Structuring medication safety narratives: development and evaluation of the medication-related incident reports annotation scheme
Source: Front Digit Health. 2026 Apr 2;8:1712526. doi: 10.3389/fdgth.2026.1712526 (PMC13083120; doi:10.3389/fdgth.2026.1712526)
Supplement: Supplementary file 1 [file Table1.docx]

**Table S1: Named entity types in the MRIRA scheme**

| **Entity type** | **Description** | **Example** | **Example from the study dataset** (with incident report code) |
| --- | --- | --- | --- |
| *Drug name* | The name by which a drug is identified, including its generic name and brand name.  Include any additional references related to drugs. | Methylphenidate hydrochloride or Concerta XL.  CDs, medication or supplies. | “***Gabapentin*** *300mg capsules TDS were dispensed into the patient’s blister-pack instead of* ***Gabapentin*** *100mg capsules TDS.*” (CD1286)  “***Pain medication*** *was prescribed on the ward*.” (CD1290) |
| *Drug strength or amount* | Drug strength definition: The concentration or potency of a drug, typically indicated by the amount of active ingredient present in a specific volume or weight of the dosage form.  Drug amount definition: The total quantity of medication prescribed for a specified period or dosage regimen, taking into account both the dosage and frequency of administration.  Additionally, the definition includes the amount of medication in bulk units, excluding if these numbers are related to a single drug dose. | Product concentration (e.g., 30mg).  Drug amount (e.g., 3 ampules, 3 tablets, 30 ml, or 3 bottles).  Bulk unit (e.g., tablets, ampules or bottles). | “*Theatre 1 ordered* ***20 vials*** *of morphine* ***10mg/ml*** *injection*.” (CD1289) |
| *Drug form* | The physical form of a dose of medication, which may include tablets, capsules, syrups, injections, patches, etc., designed for administration to a patient.  Additionally, the definition includes formulations-related entities. | Capsule, vial, tablet, ampule or any related word such as syringe.  Extended-release or long-acting tablets. | “*Gabapentin 300mg* ***capsules*** *TDS were dispensed into the patient’s blister-pack instead of Gabapentin 100mg* ***capsules*** *TDS*.” (CD1286) |
| *Drug dose* | The amount of medication (usually expressed in milligrams or millilitres) to be taken at one time or over a specified period. | 20mg or 20ml. | “*Gabapentin* ***300mg*** *capsules TDS were dispensed into the patient’s blister-pack instead of Gabapentin* ***100mg*** *capsules TDS*. “ (CD1286) |
| *Dose frequency* | The number of times a medication is taken within a given timeframe, indicating the intervals between administrations or the time of administration. | Once daily, twice daily, every 8 hours, at bedtime, am. | “*Gabapentin 300mg capsules* ***TDS*** *were dispensed into the patient’s blister-pack instead of Gabapentin 100mg capsules* ***TDS***.” (CD1286)  *On admission, she was prescribed a* ***stat*** *dose of IV paracetamol*. (CD1290) |
| *Dose route* | The path by which a drug or other substance is taken into the body. | Orally (by mouth), intravenously (into a vein) or topically (on the skin).  Alternatively, any related word such as syringe. | “*She was prescribed a stat dose of* ***IV*** *paracetamol*.” (CD1290) |
| *Dose duration* | The period over which a specific dosage regimen is prescribed or recommended for the treatment of a medical condition, indicating the duration of therapy.  Additionally, the definition includes the count of doses for planned or already administered therapy. | 2 weeks, 14 days or 3 doses. | None |
| *People* | It refers to human beings collectively, including men, women, and children.  The definition includes patients and any healthcare providers (irrespective of whether or not they are involved in the incident), detailing their roles and titles.  Furthermore, it includes any other individuals mentioned in the report. | Nurses, pharmacists, and GPs.  Family members, friends, neighbours, colleagues or caregivers. | “*I discussed the error with the* ***patient’s son*** *11/8/21 who advised* ***me*** ***he*** *had not given* ***his mother*** *any of the new meds and had actually used an old supply.*” (CD1286)  “*The* ***patient*** *had been given a morning dose of 300mg Gabapentin in error by the* ***carers***.” (CD1286)  “*The incident was discussed with the* ***medical team****.* ***Patient*** *was advised to omit next two doses of gabapentin*.” (CD1286)  “*Reflective learning undertaken by the* ***dispenser*** *and the* ***checker****.*” (CD1286)  “*3 year old* ***child****. Fall around 2.5m onto hard surface.*” (CD1287) |
| *Location* | The geographical position or place where something exists or can be found. | Home care facility, hospital, ambulance, ward, ER, operation room, clinic or GP practice. | “*The error was discovered 11/8/21 in the afternoon after the patient had gone* ***home***.” (CD1286)  “*Pain medication prescribed on* ***ward***.” (CD1290) |
| *Artefact* | Refers to any tangible or electronic tools or instruments. | Drug book, medical record, ambulance. | “*Gabapentin 300mg capsules TDS were dispensed into the patient’s* ***blister-pack*** *instead of Gabapentin 100mg capsules TDS*.” (CD1286)  “*I discussed the error with the patient’s son 11/8/21 who advised me he had not given his mother any of the* ***new meds*** *and had actually used an* ***old supply****.*” (CD1286)  “*When dispensing and checking ensure the drug name, strength, form and dose on the* ***prescription*** *matches what is dispensed and what is labelled*.” (CD1286)  “*3 year old child. Fall around 2.5m onto* ***hard surface***.” (CD1287) |
| *Knowledge* | Refers to entities related to brain function.  Also, in our guidelines, knowledge entity also includes any term used to describe something governed by reason or evidence. | Observation, calculation, awareness.  Error or wrong dose. | “*I discussed the* ***error*** *with the patient’s son 11/8/21 who advised me he had not given his mother any of the new meds and had actually used an old supply.*” (CD1286) |
| *Function* | Function refers to any other important entities related to physical processes. | Clerking, training, discharge. | “***Reflective learning*** *undertaken by the dispenser and the checker*.” (CD1286)  “*When* ***dispensing*** *and* ***checking*** *ensure the drug name, strength, form and dose on the prescription matches what is dispensed and what is labelled.*” (CD1286) |
| *Date or time* | The progression of events from the past through to the present and future, measured in seconds, minutes, hours, days, months or years, often exploring temporal relationships, sequences, and patterns. | July 14^th^, 2023, 12 days ago, 12 am, last week, next day, morning or night shift. | “*The error was discovered* ***11/8/21*** *in the* ***afternoon*** *after the patient had gone home*.” (CD1286)  “*On* ***admission****, she was prescribed a stat dose of IV paracetamol*.” (CD1290)  “*3 year old child. Fall around* ***2.5m*** *onto hard surface.* “ (CD1287) |
| *Medical condition* | A health condition, disease or disorder for which a medication is prescribed or indicated to alleviate symptoms, manage the condition or achieve therapeutic outcomes.  Additionally, the definition includes references to death if it is relevant to the incident report or narrative text being annotated. If a patient’s death is mentioned in the context of the incident report, it would be annotated as part of the medical condition type. | Diabetic patient, high blood pressure, elevated heart rate or tachycardia.  Death, no response. | “*A patient was admitted under the trauma and orthopaedic team with a* ***fractured pubic ramus***.” (CD1290) |
| *Age group*:   - Paediatric age ranges*: Neonates (0-28 days), infants (29 days to 2 years), toddlers (2-5 years), and children (6-17 years).* - Adult age ranges: *Young adults (18-35 years), adults (36-64 years)*. - Geriatric age ranges: *Young-old (65-74 years), old (75-84 years), and very old (85+ years).* | | 45 years old | “***3 year*** *old child. Fall around 2.5m onto hard surface*.” (CD1287) |
| *Gender*:   - *Female* - *Male* - *Other* | Gender refers to the social and cultural expectations, roles, and behaviours associated with being male, female or another gender identity in a particular society or community.  Additionally, the definition includes any expression identifying the patient’s gender such as ‘her’ or ‘him.’ | Male or female.  Her, his, he or she. | “*I discussed the error with the patient’s son 11/8/21 who advised me* ***he*** *had not given his* ***mother*** *any of the new meds and had actually used an old supply*.” (CD1286) |
