# Supplementary material for: Structuring medication safety narratives: development and evaluation of the medication-related incident reports annotation scheme
Source: Front Digit Health. 2026 Apr 2;8:1712526. doi: 10.3389/fdgth.2026.1712526 (PMC13083120; doi:10.3389/fdgth.2026.1712526)
Supplement: Supplementary file 2 [file Table2.docx]

**Table S2: Event types of the MRIRA scheme**

| **Event type** | **Definition** | **Possible arguments** | **Example from the study dataset** (with incident report code) |
| --- | --- | --- | --- |
| *Prescribing* | The process whereby a healthcare provider (e.g., physician, nurse practitioner) decides to recommend and authorise the use of a medication for a patient based on their diagnosis and medical history. | Agent: People.  Subject: Drug_name, Drug_dose, Drug_strength_amount, Artefact, Function or Knowledge.  Receiver: People.  When: Date_time.  Where: Location. | “*Phenobarbital* ***was prescribed*** *120 mg once/day. Stock dose Phenobarbital tablet 30 mg; only one tablet was taken out, hence, 90 mg was unintentionally not given.*” (CD1259) |
| *Transcription* | The process of accurately transferring the details of a prescription from a healthcare provider’s instructions into a format that can be understood and acted upon by others involved in the medication process, such as pharmacists. | Agent: People.  Subject: Drug_name, Drug_dose, Drug_strength_amount, Artefact, Function or Knowledge.  Receiver: People.  When: Date_time.  Where: Location. | “*Dose prescribed: alfentanil 4.8mg over 24 hours CD register: 4.8microgram had* ***been booked out****.*” (CD1246)  “*Medication on paper prescription chart not* ***transcribed*** *onto the electronic prescription when patient moved from ITU to ward.*” (CD1310) |
| *Dispensing* | Involves the actual preparation and packaging of the prescribed medication by a pharmacist or another authorised healthcare professional. This stage ensures that the correct medication, dosage, and instructions are provided to the patient. | Agent: People.  Subject: Drug_name, Drug_strength_amount, Artefact, Function or Knowledge.  Receiver: People.  When: Date_time.  Where: Location. | “*Patient* ***was handed*** *another patients methadone*.” (CD121) |
| *Administration* | This refers to the act of giving the prescribed medication to the patient. This may be done by the patient themselves, a caregiver or a healthcare professional, depending on the situation and type of medication. | Agent: People.  Subject: Drug_name, Drug_strength_amount, Artefact, Function or Knowledge.  Receiver: People.  When: Date_time.  Where: Location. | “*Nurse advised wrong dose of alfentanil had* ***been given*** *via syringe driver as it was made up incorrectly.*” (CD1246) |
| *Monitoring* | Involves ongoing observation and assessment of the patient’s response to the medication. Healthcare providers track the patient’s progress, monitor for any side effects or adverse reactions, and adjust treatment as necessary to ensure optimal therapeutic outcomes. | Agent: People.  Subject: Drug_name, Drug_dose, Drug_strength_amount, Artefact, Function or Knowledge.  Receiver: People.  When: Date_time. | None |
| *Corrective action* | Corrective action involves immediate post-incident measures such as holding drugs, changing drug doses, giving a specific drug or doing CPR to prevent or reduce patient harm. | Agent: People.  Subject: Artefact, Function, Knowledge, Drug_name or Drug_form.  Receiver: People.  Where: Location.  When: Date_time. | “*Pharmacy team tried to* ***contact*** *patient but was unable to reach them. Relevant* ***notes made*** *on patients PMR and drugs team* ***contacted****.*” (CD121)  “*Patient then had seizure which* ***was terminated*** *with lorazepam.*” (CD1258) |
| *Preventive action* | Preventive action includes plans or recommendations for future prevention, such as SOP reviews, system changes or staff training. | Agent: People.  Subject: Artefact, Function, Knowledge or Drug_name.  Receiver: People.  When: Date_time or Function | “*All branch colleagues* ***re-read*** *relevant SOPs to ensure that the procedures in branch are followed consistently. Any training needs that this identified should* ***be actioned****. Reiterated that CDs should* ***be double checked*** *before reaching the pharmacist as per SOP.*” (CD121) |
| *Underlying and contributing factor* | Underlying contributing factors denote fundamental elements which significantly influence main events, often as root causes. | Agent: People or Artefact,  Subject: Artefact, Function, Knowledge, Drug_name, Drug_dose, Drug_strength_amount or Medical_condition.  Receiver: People.  When: Date_time.  Where: Location. | “*One person* ***dealing with*** *the methadone dispensing and handing out.*” (CD121)  “*TTO had been done when patient was admitted 2 months prior. RMO had* ***not updated*** *TTO.*” (CD1258) |
| *Error outcome* | Error outcome refers to harm or symptoms experienced by patients, such as elevated blood pressure, uncontrolled heartbeat or necessitating hospitalisation, life-sustaining interventions or leading to death. | Agent: People, Drug_name or Artefact.  Subject: People, Artefact, Function, Knowledge, Medical_condition or Drug_name.  Receiver: People.  Where: Location.  When: Date_time. | “*Patient readmitted with accidental drug overdose.*” (CD12550)  “*Patient then* ***had seizure*** *which was terminated with lorazepam and patient* ***failed to wake up.***”(CD1258) |
| *Action taken* | Certain actions specified in the report, including “doctor was informed,” “incident was Datixed” or “error was investigated” do not align with the defined scope for corrective actions. These actions are included in ‘Action taken.’  Annotators should also label follow-up actions as ‘Other action’, like reviews or reassessments. | Agent: People or Artefact.  Subject: Artefact, Function, Knowledge, Drug_name, Drug_form, or Medical_condition.  Receiver: People.  Where: Location.  When: Date_time. | “*The Recovery Coordinator also* ***checked*** *CRiiS records and* ***spoke to*** *Admin who confirmed the script had been curtailed on 26.08.21.*” (CD990) |
| *Other action* | This includes any pre-incident actions described in the text such as “patient was admitted to hospital’” or “patient was transferred by ambulance.” | Agent: People or Artefact.  Subject: Artefact, Function, Knowledge, Drug_name, Drug_form or Medical_condition.  Receiver: People.  Where: Location.  When: Date_time. | “***Admitted*** *with seizures.*” (CD1271)  “*A patient* ***was admitted*** *under the trauma and orthopaedic team with a fractured pubic ramus.*” (CD1290) |
