# Supplementary material for: Structuring medication safety narratives: development and evaluation of the medication-related incident reports annotation scheme
Source: Front Digit Health. 2026 Apr 2;8:1712526. doi: 10.3389/fdgth.2026.1712526 (PMC13083120; doi:10.3389/fdgth.2026.1712526)
Supplement: Supplementary file 3 [file Table3.docx]

**Table S3: Event attributes of the MRIRA scheme**

| **Event attribute** | **Description** | **Example from the study dataset** (with incident report code) |
| --- | --- | --- |
| *Error: Action was intended and actual* | When the action was carried out with the intention to achieve a certain outcome. | “*Met call to patient found unresponsive, given naloxone for opioid toxicity and respiratory rate and alertness improved. Patient then had seizure which was terminated with lorazepam and patient failed to wake up. Bloods were taken after the MET call and it was identified the patient was in AKI - stage 2.*” (CD1258)  “*Patient went to theatre patient had allergies recorded to codeine and tramadol and was given morphine in theatre. Became drowsy, 2 x doses of naloxone were given to patient. patient then returned to the ward with a PCA …. Anaesthetist aware of patient’s sensitivities but this created a challenge for pain relief for a painful procedure.*” (CD1283) |
| *Error: Action was unintended but actual* | When the action or decision was not the intended course of action. | “*Dose prescribed: alfentanil 4.8mg over 24 hours CD register: 4.8microgram had been booked out Preparation used: alfentanil 1mg/2ml amp.*” (CD1246)  “*Phenobarbital was prescribed 120 mg once/day. Stock dose Phenobarbital tablet 30 mg; only one tablet was taken out, hence, 90 mg was unintentionally not given.*” (CD1259) |
| *Error: Action intention is unclear* | When the intention behind the action or decision is unclear from the incident description. | “*Discharged home form AMU home - tramadol 100mgs included on drugs to take home prescription. Tramadol should have been discontinued. Patient readmitted with accidental drug overdose.* “ (CD1255) |
| *Negated* | When segments of text referring to events are negated; indicated by phrases such as ‘not’, ‘no’ or ‘without.’ Negation labelling must be applied during the event’s annotation process. | “*TTO had been done when patient was admitted 2 months prior. RMO had not* ***updated*** *TTO.*” (CD1258)  “*I Identified bisoprolol, oxycodone MR and lidocaine patches were not* ***prescribed.***” (CD1248)  “*Phenobarbital was prescribed 120 mg once/day. Stock dose Phenobarbital tablet 30 mg; only one tablet was taken out, hence, 90 mg was unintentionally not* ***given****.*” (CD1259) |
